# Design and test of novel scent enrichments to enhance breeding of zoo-housed lemurs

**DOI:** 10.12688/f1000research.144636.1

**Published:** 2024-02-19

**Authors:** Emily Elwell, Sara Fontani, Stefano Vaglio

**Affiliations:** 1 Animal Behaviour & Wildlife Conservation Group, School of Life Sciences, University of Wolverhampton, Wolverhampton, England, WV1 1LY, UK; 2 University College–The Castle, Durham University, Durham, England, UK

**Keywords:** scent enrichment, behavioural observations, sexual behaviours, mating, faecal endocrinology, semiochemistry, gentle lemurs, ruffed lemurs

## Abstract

**Background:**

Zoos use environmental enrichments, including scents, which may have applications to improve breeding success for taxa, such as lemurs, which rely heavily on olfactory communication. We aimed to develop novel, biologically-relevant scent enrichments to trigger mating behaviours of zoo-housed lemur species, which are critically endangered in the wild and show a low success rate in captive breeding programmes.

**Methods:**

We examined anogenital odour secretions, released by female gentle ( *Hapalemur alaotrensis*) and ruffed ( *Varecia variegata*) lemurs, using solid-phase microextraction and gas chromatography-mass spectrometry techniques. We identified the key compounds distinguishing the volatile chemical profile of female lemurs during the breeding season and used them to develop species-specific scent enrichments. We then tested the scent enrichments, made up of synthesized mixtures conveying information about female lemur fertility, on unsuccessful breeding pairs of lemurs hosted in European zoos. We evaluated the effects of the newly designed scent enrichments on their target species by combining behavioural observations with faecal endocrinology.

**Results:**

We identified and reproduced fertility-specific signals associated with female scents. These scent mixtures triggered male sexual behaviours, including mating, during and after the enrichment condition. We also found effects on faecal testosterone levels, with increased levels after the enrichment condition albeit not statistically significant.

**Conclusions:**

Our findings suggest that biologically-relevant scent enrichments may trigger natural species-specific behaviours, with potential implications for conservation breeding of zoo-based endangered lemur species, and highlight that combining more assessment methods may assist with evaluating the impact of environmental enrichments.

## Introduction

Zoo populations are typically managed to provide the public with education about wildlife and their environmental challenges, and to uplift endangered species through both *ex-situ* conservation breeding and *in-situ* reintroduction programmes ( Schulte-Hostedde & Mastromonaco, 2015). In this context, the maintenance of the genetic variation of such captive populations is imperative ( Lacy, 2009). However, captive populations, potentially serving as buffers against extinction, may experience problems that impair them from being viable for reintroduction into the wild. Specifically, zoo populations may face reproductive issues which inhibit them from serving as viable ‘reserve populations’ ( Meier, 2016). Furthermore, managing zoo populations is challenging because of the mismatch between natural and captive environments ( Carroll *et al.*, 2014). Primates evolved distinct behavioural patterns, and difficulty in engaging in these behaviours can cause frustration or boredom, which, in turn, can lead to stress and development of abnormal behaviours (for around 50% of zoo animals) ( Hosey, 2005) that undermine their individual welfare and their breeding success.

To maintain captive healthy populations, with good genetic variability and thus high survival rate in case of reintroduction, modern zoos take part in conservation breeding programmes (e.g., European Association of Zoos and Aquaria (EAZA)’s *Ex situ* programmes – EEP). Moreover, as reproductive success is linked to the degree of similarity between captive environmental conditions and those that animals would experience in the wild ( Meier, 2016), zoos use environmental enrichments to improve the well-being of captive populations. Environmental enrichments and conservation breeding are closely related, as enrichment is a dynamic process that modifies an animal’s environment, prompting a wider range of species-specific behaviours ( Ben-Ari, 2001) promoting resiliency to stress, which in turn helps animals recovering from adverse stimuli ( Quirke & O’Riordan, 2011), as well as improving the exhibit from the perspective of visitors ( Carlstead & Shepherdson, 1994). Furthermore, enrichment can foster the essential abilities that animals would need for their survival if reintroduced into their natural habitat ( Rioldi, 2013). Additionally, conservation breeding programmes, through breeding management recommendations, maximize the genetic diversity and reduce the loss of genetic variation associated with genetic drift, which can be rapid in small captive populations ( Ballou *et al.*, 2010).

With almost 60% of primate species currently facing an extremely or very high risk of extinction in the wild, raising global awareness of the difficulty of the world’s primates is vital ( Estrada *et al.*, 2017). Among primates, lemurs are the most endangered taxa; around a third of the 107 species currently living in Madagascar are classified as critically endangered, while almost all of them are at risk of extinction ( IUCN, 2023). Lemurs are endemic to Madagascar, which is a recognized biodiversity hotspot and arguably the top priority for primate conservation ( Mittermeier, 2014). Moreover, several captive lemur populations are also struggling, in terms of both abundance and demographic trend, almost as much as their wild counterparts, and currently would not support reintroduction into the wild ( Meier, 2016). However, captivity, unlike the wild, is a human-controlled environment and thus it is possible to enhance captive breeding via environmental enrichment and evidence-based facilitation of breeding ( Meier, 2016).

The overarching aim of this research work was to develop new scent enrichments to enhance breeding success of zoo-housed lemurs. To achieve this, we investigated the chemical profile of the anogenital odour secretions of successful breeding females, then reproduced the chemical mixture in our semiochemistry laboratory (focusing on volatile compounds) and tested it with unsuccessful breeding pairs (i.e., biologically able to reproduce but never been successful as a breeding pair). Then, to assess the effects of the scent enrichment, we combined behavioural observations (focusing on sexual behaviours) with faecal endocrinology (focusing on sex hormones).

Specifically, we aimed to:

• Identify the key compounds that convey information about female lemur fertility.
• Design novel scent enrichments made up by species-specific chemical mixtures signalling female fertility.
• Evaluate whether the newly designed scent enrichments trigger sexual behaviours.

## Methods

This study adheres to the ARRIVE guidelines ( Vaglio, 2024).

### Ethical considerations

The use cases/studies followed the institutional and international guidelines for the care and use of captive animals, involving non-invasive methods for obtaining behavioural data, faecal and odour samples from the lemurs. Moreover, the studies were conducted in compliance with the Convention on Biological Diversity and the Convention on the Trade in Endangered Species of Wild Fauna and Flora and approved by the Life Sciences Ethics Committee (LSEC) at the University of Wolverhampton (UK) (REC numbers LSEC/201819/CY/166 and LSEC/202021/SV/52) and the Ethics Committees at Jersey Zoo (Channel Islands), Parc Zoologique & Botanique de Mulhouse (France), Birmingham Wildlife Conservation Park, Dudley Zoo & Castle, Shaldon Zoo – Wildlife Trust, Twycross Zoo, and ZSL London Zoo (UK). The authors made all possible efforts to ameliorate harm to animals, which was achieved by using non-invasive methods to collect samples (including behavioural data and biological samples such as anogenital odour secretions and faeces) from the study subjects.

### Study subjects and housing

We studied four pairs of gentle lemurs ( *Hapalemur alaotrensis*) (N = 8), hosted at Birmingham Wildlife Conservation Park (UK), Parc Zoologique & Botanique de Mulhouse (France), Jersey Zoo (Channel Islands) and ZSL London Zoo (UK) ( Fontani *et al.*, 2022), and four small groups of red ruffed and black-and-white ruffed lemurs ( *Varecia* spp.) (N=15) at Dudley Zoo & Castle, Shaldon Zoo – Wildlife Trust, and Twycross Zoo (UK) (Elwell *et al.*, unpublished data). All study troops were housed in indoor enclosures (heated to 25-28°C) and had access to outdoor enclosures.

### Study protocol

We divided the study period into three phases: pre- enrichment (i.e., before enrichment condition – two weeks), enrichment (i.e., during enrichment condition – one week), post- enrichment (i.e., after enrichment condition – two weeks). We carried out behavioural observations and faecal sampling every study day from early morning to early afternoon, as they are more active in the morning (~8AM-1PM, 5 hours per day), over five days per week. We assessed the effects of the enrichment combining the observation of sexual behaviours (including mating) and faecal endocrinology (e.g., faecal testosterone levels in males).

### Odour sampling and investigation

We collected anogenital odour samples by rubbing 10 times a sterile cotton swab around the wall of the vulva, using steady pressure, as described by Vaglio *et al.* (2021a). Moreover, we exposed control swabs to the air to identify any compounds that did not derive from the lemurs. We placed all samples and controls into sterile vials and immediately stored them in a −20°C freezer at the zoo. We then transferred the vials to the Rosalind Franklin Science Centre, University of Wolverhampton, using a freezer box with ice packs to avoid any risk of defrosting, for laboratory analyses.

We investigated the volatile component of odour signals using solid-phase microextraction (SPME) and gas chromatography-mass spectrometry (GC-MS) techniques, as described by Walker & Vaglio (2021). Briefly, we introduced a 65 μm polydimethylsiloxane/divinylbenzene SPME syringe needle through the vial septum and exposed the fibre to the headspace above the sample in the vial for 15 min at 40 °C. We analysed the adsorbed volatile analytes of all samples using a 5975C mass spectrometer (Agilent Technologies) EI, 70 eV, coupled directly to a 7890B gas chromatograph (Agilent Technologies) equipped with a fused silica HP5-MS UI capillary column (Agilent Technologies) 30 m × 0.25 mm crossbonded 5%-phenyl-95% dimethylpolysiloxane, film thickness 0.25 μm. We maintained the injector and transfer line temperatures at 270 °C and 280 °C, respectively. We made injections in splitless mode (purge valve opened after 1 min) with a constant flow of helium carrier gas of 1 ml/min. We started the oven temperature programme at 45 °C for 2 min, then raised it by 4 °C /min to 170 °C, and finally by 20 °C/min to 300 °C 40.

We assessed possible environmental contamination via blank analyses using an empty 10 ml vial (Supelco) and control swabs following the same procedure as for the samples and conditioned the fibre at 260 °C pre-injection for 5 min and 260 °C post-injection for 20 min to avoid any possible carry-over effects. We analysed all samples in a short period of time to minimize inter-assay variability. We overlaid chemical profiles from control swabs on lemur chemical profiles to identify compounds that did not derive from the lemurs and removed these from the swab results.

We tentatively identified eluted compounds by comparing the experimental spectra with those of the mass-spectral library in ChemStation (Agilent Technologies) and NIST Database (National Institute of Standards and Technology), version MSD F.01.01.2317 (Agilent Technologies). We accepted a putative identification if the minimum matching factor was higher than 90%. After that, we carried out the unequivocal identification of the key compounds distinguishing the fertile window of the breeding female comparing these compounds with standard compounds injected and analysed by applying the same SPME and GC-MS protocol (Elwell *et al.*, unpublished data).

### Scent enrichment

Briefly, we diluted each chemical compound separately, placing 1.5 mL of HPLC grade methanol (Fisher Chemical, Cat. number 10499560) in 15 mL test tube, adding 5 μL of compound and 3.5 mL of de-ionised water, and then we vortexed for 15 seconds to dissolve the compound in the mixture. We compared both the retention times of key compounds and standards and the overall patterns of the mass spectra. We accepted the identification only if both the parameters were satisfied. Once the identification was certain, we added 1 mL of each diluted compound into a new test tube and vortexed for 30 second to produce the scent mixtures to test as olfactory enrichment.

**Figure 1.**
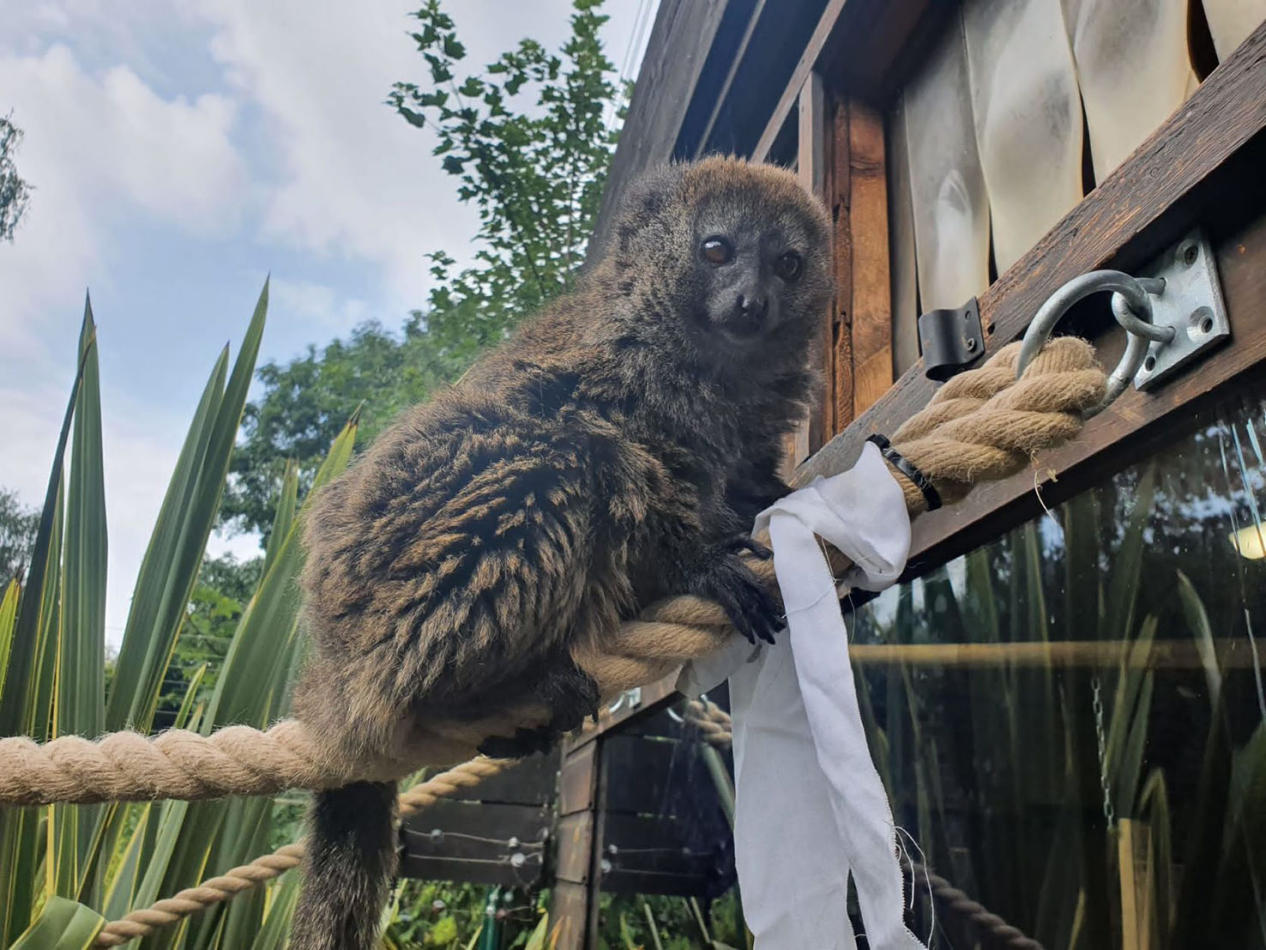
Male gentle lemur interacting with scent enrichment in the outdoor enclosure at Birmingham Wildlife Conservation Park. Photo by Georgia Callagan with permission.

We then presented the enrichment to the study subjects applying the protocol described in Vaglio *et al.* (2021b). Briefly, we used white cotton sheets cut into 75 cm long and 5 cm wide strips, which were soaked with 20 drops of scent mixture diluted with 12 ml of cold boiled water. Newly soaked cotton strips were prepared each enrichment day. We placed 2 unscented (controls) and 6 scented strips on the climbing frames both indoor and outdoor ( Figure 1) and removed them at the end of observations every study day. To avoid habituation, we randomized the locations of both scented and unscented cotton strips daily.

### Behavioural data collection

We collected behavioural data using all occurrences of some behaviours, instantaneous scan, and *ad libitum* sampling methods ( Altmann, 1974) and focused on sexual behaviours ( Table 1). For each study subject, we determined the relative frequency of each behavioural category, calculating the number of behaviours performed out of the total hours of observation.

**Table 1.** Ethogram ( Fontani *et al.*, 2022; Elwell *et al.*, unpublished data).

Behaviour	Description
Sniffing/Licking Conspecific Genitals	An individual deliberately places their nostrils/tongue within 3 cm from a conspecific and sniffs/licks. Primarily sniffing of the genital area but may include other parts of body.
Mounting	Attempt mounting – The male approaches the female, clasps, orients body for copulation. The female chatters at and/or cuffs the male, and the male releases the female. Mounting with copulation – The male mounts on top of the female and thrusts. The male introduces sperm into the female reproductive tract.
Solicitation	Squeal approach – The male advances submissively towards the female in a quivering crawl. Head may be extended, and ears flattened. The male will emit a roar-shriek noise and the female will also make this noise in chorus. Suspension – The male suspends himself beneath the female and makes a chattering noise/acts submissive. His head may be extended and ears flattened. Follow – The male approaches the female from behind and follows closely.
Penile erection Mating calls	The male shows a conspicuously erect red penis. The female produces distinct single or series of calls, while soliciting copulation and during mating.

We performed the inter-observer reliability test to measure the degree of agreement in the behaviour identification by the different observers at the zoo facilities ( Wark *et al.*, 2021). Specifically, we used Cohen’s Kappa coefficient to measure the agreement between the observers.

### Faecal hormone sampling and measurements

We collected faecal samples every morning during study days (i.e., when behavioural observations were conducted), right after defecation was observed, when the identity of the study subject was certain. As diurnal secretion patters of hormones, such as testosterone, may be detected in faecal samples (especially for small-bodied species), we restricted the sampling period to approximately the same time of the day ( Hodges & Heistermann, 2011). We stored the samples in a −20 °C freezer on site immediately after sampling. At the end of the study period, we transferred the samples to the Rosalind Franklin Science Centre – University of Wolverhampton using a cold bag with ice packs to avoid any risk of defrosting.

*Hormone analyses*

We used a freeze-drying machine (Beta 1–8 LSC plus, Christ R) to lyophilize the faecal samples for 72 h, and then we pulverized them using a pestle and mortar. We sieved the faecal powder through a stainless-steel strainer, aperture 250 mic, to separate the faecal residue from any fibrous material. With regards to extraction, we followed the methods described in Fontani *et al.* (2022). Briefly, we extracted 0.05–0.1 g of faecal powder in 3 ml of 80% methanol (Scientific Laboratory Supplies, Cat. number CHE2536) using a 15 ml plastic tube and vortexing it for 15 min with a multi-tube vortexer (Multi-Vortexer V-32, Grant Instruments R). Right after centrifugation for 20 min at 3,300 ×g, we stored the supernatant at −20 °C.

When analysing faecal hormones, we considered the time course of hormones metabolite excretion relative to the production and circulation of the native hormones ( Hodges & Heistermann, 2011; Wheeler *et al.*, 2013). We measured faecal testosterone levels using commercially available enzyme-linked immunosorbent assay (ELISA) kits (DetectX ^®^ Testosterone K032-H5W, Arbor Assays R, USA, Cat. number K032-H5) following kits instructions. Before analysis, we diluted all the samples 1:1 with the assay buffer provided by the kits. We assayed all standards and faecal samples in duplicates, with samples showing a coefficient of variation (CV) exceeding 15% being re-analysed ( Macagno *et al.*, 2020). We analysed assay data applying a 4-parameter logistic fitting programme (MyAssays R, – open access, available online at https://www.myassays.com/index.html). Concentrations were expressed as pg/mg. Mean intra-assay coefficient of variation for testosterone, tested on three control samples (all males), was 9.35% ± 2.57. Mean inter-assay coefficient of variation, tested on the same samples measured with four replicates across three assay plates, was 5.96% ± 1.42 for testosterone.

## Use cases

The above described methods have been previously implemented in our studies (odour sampling: Vaglio *et al.*, 2021a; odour investigation: Walker & Vaglio, 2021; scent enrichment protocol: Vaglio *et al.*, 2021b; behavioural and endocrinological data collection: Maréchal *et al.*, 2011; Fontani *et al.*, 2022; Vaglio *et al.*, 2021b).

As case studies to show how the methods are expected to be implemented by the research community, we report the results that we obtained with regards to gentle lemurs ( Fontani *et al.*, 2022; Fontani *et al.*, unpublished data) and ruffed lemurs ( *Varecia* spp.) (Elwell *et al.*, unpublished data).

### Gentle lemur case study – Odour results

**Figure 2.**
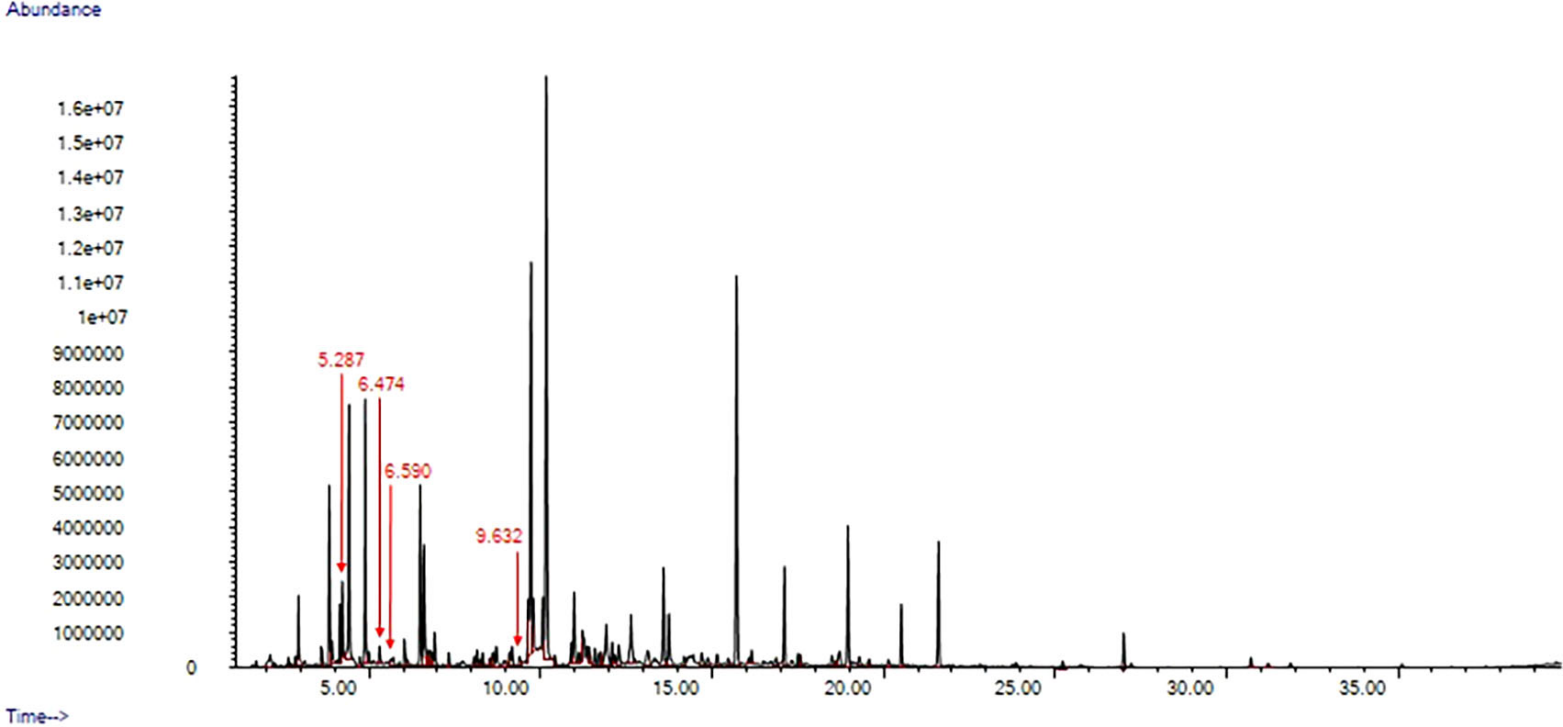
Example chromatogram from female gentle lemur ( *Hapalemur alaotrensis*), anogenital odour sample from fertile period. The peaks of the key compounds are pointed out with a red arrow.

We unequivocally identified four compounds (2-heptanone; 3-heptanone; 3-octanone; 4-methyl 3-hexanone) that were only present in the chemical profiles of anogenital odour samples collected during the fertile window of the breeding period. A representative chromatogram from the fertile window is shown in Figure 2.

### Gentle lemur case study – Behavioural results

The frequency of male sexual behaviours significantly increased (β ± SE = 0.605 ± 0.211, t-value= 2.865, p-value = 0.0046) during the enrichment condition compared to the pre-enrichment condition. The post-enrichment condition also showed a higher frequency of sexual behaviours than the pre-enrichment condition with a significant tendency (β ± SE = 0.323 ± 0.184, t-value = 1.754, p-value = 0.081).

### Gentle lemur case study – Endocrinological results

We did not find any significant relationship between faecal testosterone levels and the study period (p-value > 0.05).

### Ruffed lemur case study – Odour results

**Figure 3.**
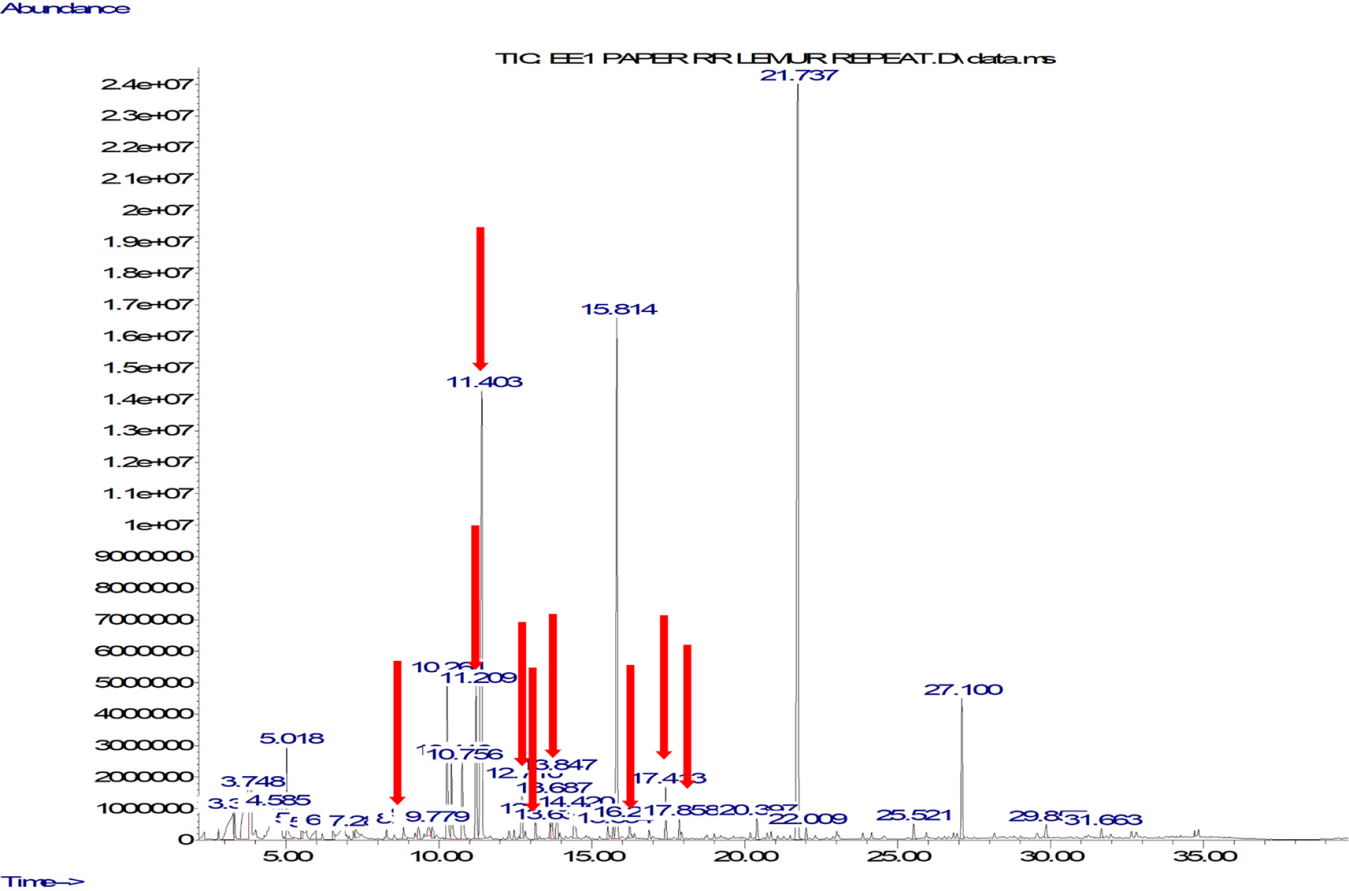
Example chromatogram from female red ruffed lemur ( *Varecia variegata rubra*), anogenital odour sample from breeding period. The peaks of the key compounds are pointed out with a red arrow.

We unequivocally identified 12 compounds (benzaldehyde; 1-hexanol, 2-ethyl; benzyl alcohol; dihydromyrcenol; 1-octanol; 2-phenyl-2-propanol; tetrahydrolinalool; linalool; nonanal; menthol; decanal; 2-phenoxyethanol) as being key to the breeding period (i.e., including compounds present only in the breeding period and compounds with much higher relative abundance over the breeding period) in the chemical profiles of anogenital odour samples collected during the breeding period. A typical chromatogram from the breeding period is shown in Figure 3.

### Ruffed lemur case study – Behavioural results

**Figure 4.**
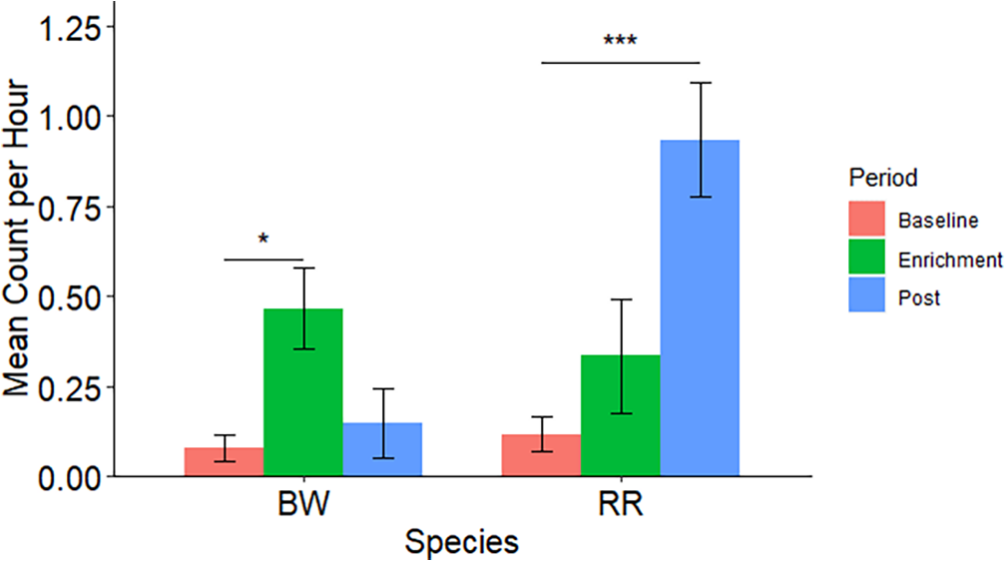
The mean count per hour of sexual behaviours by male ruffed lemurs ( *Varecia* spp.) during each study period. BW indicates black-and-white ruffed lemurs ( *Varecia variegata variegata*); RR indicates red ruffed lemurs ( *Varecia variegata rubra*). A * indicates a significant difference (*: P = 0.05-0.01; **: 0.01 > P = 0.001; ***: P < 0.001).

There was a significant effect of period on male sexual behaviours. Ruffed lemurs increased their sexual behaviours while the scent enrichment was present (Est = 2.069; SE = 1.008; z = 2.052; P = 0.040). In comparison, sexual behaviours continued throughout the study after scent exposure and were highest during the post-enrichment period (Est = 1.931; SE = 0.486; 3.978; P < 0.001) ( Figure 4).

We found that mating behaviours specific to the breeding season were triggered in males once exposed to the scent enrichment. Overall, there was a significant effect of period on mounting in males and this was highest while the enrichment was present (Est = 2.998; SE = 0.817; z = 3.671; P < 0.001; R2 = 0.228).

### Ruffed lemur case study – Endocrinological results

**Figure 5.**
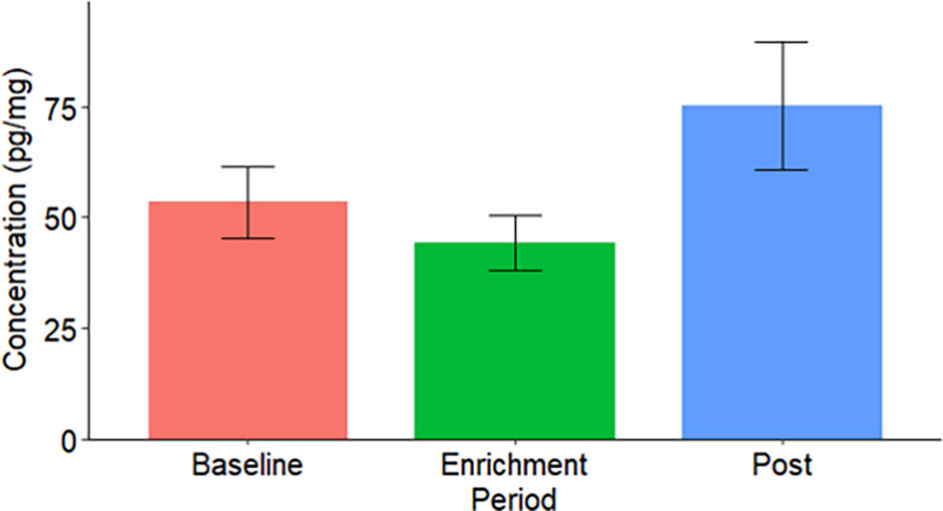
The mean faecal testosterone concentration (pg/mg) of ruffed lemurs ( *Varecia* spp.) during each study period. BW indicates black-and-white ruffed lemurs ( *Varecia variegata variegata*); RR indicates red ruffed lemurs ( *Varecia variegata rubra*). A * indicates a significant difference (*: P = 0.05-0.01; **: 0.01 > P = 0.001; ***: P < 0.001).

We found a large, but not significant (p-value > 0.05), increase of mean faecal testosterone concentration in the post-enrichment condition following scent exposure ( Figure 5).

## Discussion

Applying our semiochemistry methods ( Walker & Vaglio, 2021) we found a pool of volatile chemical compounds distinguishing the chemical profile of anogenital odour secretions released by female lemurs during the breeding period, suggesting that there might be fertility-specific signals associated with female scents. After that, we tested our novel scent mixture ( Fontani *et al.*, 2022; Elwell *et al.*, unpublished data) and showed that it triggered sexual behaviours, including mating, but no statistically significant effects were found on male faecal testosterone concentration.

Our findings suggest that biologically-relevant scent enrichments may trigger natural species-specific behaviours. Novel scent enrichments have, therefore, the potential to impact on captive management and conservation breeding of endangered lemur species. These results also highlight that combining more assessment methods (behavioural observations and faecal endocrinology) may assist with evaluating the impact of environmental enrichments (although it often proves difficult to find statistically significant changes in faecal hormone levels due to confounding variables).

We, however, must acknowledge some limitations that could have impacted the use cases. First, we focused on a relatively small sample size. Then, due to the small pool of odour samples, it is challenging to mix the compounds in proportions that reflect exactly the real ratios of the anogenital odour secretions released by the fertile female lemurs. Thus, we aim to conduct further investigations on the chemical profile of the female odour secretions (including non-volatile compounds) and expand the sample size when testing the mixture of compounds conveying information about female fertility (including several unsuccessful breeding groups hosted in various institutions).

## Data Availability

Open Science Framework: Design and test of novel scent enrichments to enhance breeding of zoo-housed lemurs. https://doi.org/10.17605/OSF.IO/W35YZ ( Vaglio, 2024). This project contains the following:
- Behavioural and endocrinological datasets - Completed ARRIVE checklist Data are available under the terms of the Creative Commons Zero “No rights reserved” data waiver (CC0 1.0 Public domain dedication).
