# Supplementary material for: Design and test of novel scent enrichments to enhance breeding of zoo-housed lemurs
Source: F1000Res. 2024 Feb 19;13:123. [Version 1] doi: 10.12688/f1000research.144636.1 (PMC11226944; doi:10.12688/f1000research.144636.1)
Supplement: Peer review history

# Reviewer report: Reviewer response for version 1

**DOI:** 10.5256/f1000research.158460.r255000

**Published:** 2024-07-05

**Authors:** Kerry Perkins

**Affiliations:** 1 Sparsholt University Centre, Sparsholt, UK

Overall an interesting topic presented. The justification of why the research was conducted was well established within the introduction. Potentially giving a greater explanation on how chemical signals play a role in reproductive success would lead into the specific aims outlined at the bottom of the introduction.

 The method section was very detailed which is key with a novel or new methodology. The main concern within the methodology is the repetition of 'we' at the beginning of nearly every sentence. This made reading feel a bit repetitive due to lack of variation. It would be worth to revisit the methodology section and possibly vary the sentences more to give a better flow within the section.

 Within the results section using subheadings and separation of the two species allowed for clear demonstration of the findings.

 In the discussion potential considerations could have been given to whether the faecal samples methodology gave sufficient sampling considering the change in behaviour within the males. But limitations within the studied were outlined well and future research discussed.

Is the rationale for developing the new method (or application) clearly explained?

Yes

Is the description of the method technically sound?

Yes

Are the conclusions about the method and its performance adequately supported by the findings presented in the article?

Partly

If any results are presented, are all the source data underlying the results available to ensure full reproducibility?

Yes

Are sufficient details provided to allow replication of the method development and its use by others?

Yes

Reviewer Expertise:

Wildlife ontogeny and captive reproductive cues

I confirm that I have read this submission and believe that I have an appropriate level of expertise to confirm that it is of an acceptable scientific standard.

# Reviewer report: Reviewer response for version 1

**DOI:** 10.5256/f1000research.158460.r285348

**Published:** 2024-07-04

**Authors:** Marlen Kuecklich

**Affiliations:** 1 University of Leipzig, Leipzig, Germany

The present study initially identified olfactory fertility cues in females through chemical analyses. Building on this, the study tested whether a synthesised reproduction of this scent mixture is suitable for stimulating male reproductive behaviour. This is a very interesting study, the results of which could have a real impact on the management and breeding of endangered species.

 Overall, the study is very clearly written, the study design is coherent, and the work has academic merit. However, some methodological information is missing, which would make the study fully comprehensible and allow replication by others. The description of the statistical analysis is completely missing and needs to be added. In the results section, a few more details (complete graphical representation, exact values of non-significant results) could make the interpretation of the results more comprehensible. For the behavioural and endocrinological data, all original data are available. For the chemical part, the underlying data are missing; raw data should be added here (analogous to the other parts, Excel tables with the intensities of the identified peaks per file would be sufficient). The conclusions adequately describe the results but should include a better integration of the findings into existing literature and potential applications. Below, I provide further details on which contents should be added to round off the study and make it a valuable and comprehensible suggestion for using olfactory enrichment for captive animals.

Introduction:

 The first paragraph of the introduction provides many relevant background details, but the thread through the various arguments is somewhat missing. Fundamentally, two main problems should be highlighted more clearly: 1) maintaining genetically diverse reserve populations and 2) creating conditions that allow the animals to exhibit as natural behaviour as possible. If the latter is not achieved, there is a risk of frustration and boredom (the causality here could be better formulated) and the problem that such animals might not survive in their natural habitat (this aspect is missing entirely and is only briefly mentioned in the second paragraph).

 The second paragraph of the introduction nicely highlights the influence of husbandry conditions on zoo populations. The last sentence on the impact of breeding management recommendations should be moved to the beginning of the paragraph, directly after the introductory sentence about breeding programmes. Additionally, the question arises as to whether there are already study results showing that improved husbandry conditions have a positive impact on reproductive success. If so, this would nicely follow the other cited studies.

 The third paragraph summarises the necessity of the study based on the threat status of primates. A more current reference on the overall threat status of all primate species would be beneficial. The approximately 60% from the 2017 publication has surely been exceeded by now, and a more up-to-date status (perhaps based on current IUCN data) would be nice, although the urgency is already clear.

 The last paragraph of the introduction clearly outlines the study's objectives. Here, the use of scent in enrichment is specifically mentioned the first time, and it would be beneficial to provide a brief introduction to this topic, including some examples of how scent is or can be used for animal enrichment and how it can positively influence behaviour.

Methods:

 The methods are detailed and comprehensible, although some details are missing that would be necessary for a study's replication. I have some additional questions:

 - Was there a randomised distribution of different observers (i.e., were the same pairs observed by multiple observers, and did each observer watch multiple pairs)?

 - What were the results of the inter-observer tests?

 - Were the behavioural observations conducted 'blind' to the condition and study objective, or were the observers informed about the study's aim and the condition being observed (before enrichment, during enrichment, after enrichment)?

 - What were the reasons for setting the enrichment condition to one week (shorter than the other two conditions)?

 - What were the reasons for using cotton swabs for collecting scent samples? The introduction states that the study focused primarily on volatile substances. However, cotton swabs collect significantly fewer volatile substances than, for instance, thermal desorption tubes (see our study, Kücklich et al. 2017 (Ref 1) , even though a different extraction method was used, the pattern should be similar). By choosing this method, a substantial portion of the volatile range of chemical profiles, which this study is interested in, might have been missed. A justification for the choice of adsorption medium in the methods section and a discussion on how the choice of cotton swabs might have influenced the study would be desirable.

 - How were the cotton swabs prepared/cleaned before sampling?

 - How long were the samples stored from collection to analysis in the laboratory?

 - Were the scent samples taken from the same pairs that were also observed in the behavioural observations of the "unsuccessful breeding pairs"? Based on the introduction, I would have expected scent samples from "successful" breeding animals and the presentation of synthesised scent mixtures to be shown to the "unsuccessful breeding pairs." Why was this not done (if I did not miss this detail)?

 - Which version of the NIST database was used?

 - How were the "key compounds distinguishing the fertile window of the breeding female" statistically determined?

 - Why was 1 mL of each substance used for the scent mixture instead of adjusting the concentrations to match the proportions in the analysed scent samples (surely some were significantly more/less intense than others)?

 - From which phases of the cycle were the samples of females taken (the results only state "during the fertile window of the breeding period"), and how was the fertile window determined? - The description of the statistical analysis is completely missing and needs to be added.

Results:

 The results section is clear and comprehensible, but a few more details should be added:

 - Were there no compounds with significantly higher relative abundance during the breeding period in gentle lemurs, similar to those considered in ruffed lemurs?

 - How was it statistically determined which compounds had significantly higher relative abundance during the breeding period, or what threshold was used for this?

 - The term "significant tendency" is not appropriate for a p-value of 0.081. It seems acceptable not to draw a strict black-and-white line at 0.05 and speak of a "tendency" here, but by definition it is not "significant".

 - Why is there no graphical representation of the mean count per hour of sexual behaviours for the gentle lemurs, as for the ruffed lemurs? This would help readers better understand the statistical results.

 - Why are there two different subtypes of ruffed lemurs mentioned in the plot, but the behavioural results are described together in the text? This needs better explanation and alignment (already in the methods, but also here between the description and the plot).

 - For the endocrinological results of the ruffed lemurs, the term "large, but not significant" difference is used. The magnitude of the result is unclear to the reader as only "p-value > 0.05" is provided; a complete presentation of the results with exact values would be desirable.

Discussion:

 The discussion summarises the results clearly and provides some suggestions for future studies. In the second paragraph, the first sentence states that the study triggered "natural species-specific behaviours." This should be more specifically related to sexual behaviours because there are many other natural behaviours one could consider that were not the focus of the study. Proper integration of the results into existing literature is completely missing, and a few discussion points are also lacking. For example:

 - Is it surprising that the compounds related to the fertile window of the breeding period differed completely between the two study species with no overlap?

 - Have these compounds been found in other studies on primates or mammals in relation to reproduction?

 - After what period of time would a change in male faecal testosterone concentration be expected?

 - Are there other studies to compare with?

 - What would be the recommendation on how other animal facilities could use these findings?

 - Can the results likely be generalised or applied to other species and settings?

Minor comments:

 - At the end of the description of the GC-MS program, there is a '40' that does not belong there.

 - There are two different spellings of "ml"/"mL" in the manuscript that should be standardised.

 Can the authors be sure, that this special compound mixture evoked the behavioural changes? The authors mentioned some blanks, but did not discuss them. Moreover, there was no classical control condition with having only stripes with random odour substances not being the compound mixture being important for reproduction identified in the chemical analyses and observing potential behavioural changes to them.Theoretically, any compound mixture could evoke behavioural changes just because of the additional enrichment and ideally this would be tested in a control condition comparison as well.

Is the rationale for developing the new method (or application) clearly explained?

Yes

Is the description of the method technically sound?

Partly

Are the conclusions about the method and its performance adequately supported by the findings presented in the article?

Yes

If any results are presented, are all the source data underlying the results available to ensure full reproducibility?

Partly

Are sufficient details provided to allow replication of the method development and its use by others?

Partly

Reviewer Expertise:

Behavioural ecology, olfactory communication

I confirm that I have read this submission and believe that I have an appropriate level of expertise to confirm that it is of an acceptable scientific standard, however I have significant reservations, as outlined above.

## References

[1] : Different methods for volatile sampling in mammals. *PLoS One* .2017;12(8) : 10.1371/journal.pone.0183440 e0183440 10.1371/journal.pone.0183440 28841690 PMC5571906

# Reviewer report: Reviewer response for version 1

**DOI:** 10.5256/f1000research.158460.r255001

**Published:** 2024-04-29

**Authors:** Lisa Clifforde

**Affiliations:** 1 The Zoological Society of London, London, England, UK

Elwell *et al.* present a study on the application of biologically relevant scent based enrichment to increase the occurrence of sexual reproduction behaviours in two species of zoo housed lemurs. This subject area of scent based enrichment is understudied within zoos, as is the use of enrichment to facilitate breeding, therefore the study has useful application within the field.

 The introduction provides an explanation as to the need for captive breeding programmes, and the more specific issues facing lemurs. They also discuss the challenges to successful captive breeding programmes and discuss why this area merits further investigation. However, the authors cite Hosey (2005) alongside the claim that 50% of zoo animals exhibit abnormal behaviours. This is a misrepresentation of this paper, in that it only discusses primates, not all zoo housed animals, and also does not contain any quantified figures as to the prevalence of abnormal behaviours. The link between the use of enrichment to further the aim of the study is not well explained, and the referenced paper by Meier (2016) does not cover the subject area, beyond a brief mention of appropriate light cycles. Furthermore, the rationale to trial biologically relevant scent based enrichments is also not explained or developed, leading the reader to wonder why this area of enrichment was decided upon. The introduction would benefit from a discussion of enrichment, including a definition (given the wide variation of interpretations in use), and why the authors hypothesised scent would aid in captive breeding attempts.

 The technical methodology of extracting odour samples and faecal hormone sampling is not with the scope of the reviewer to evaluate. The study protocol describes a two week before enrichment condition, a one week during enrichment condition, and a two week post enrichment condition, but the difference in condition durations is not explained. An explanation as to why faecal testosterone was chosen as a measurable parameter is absent from the text. A brief note as to why this is the most relevant indicator would be helpful.

 The discussion is notably brief and does not consider why the use of scent elicited the observed behaviours, or how the enrichment could be utilised in the longer term, or wider context to benefit conservation breeding programmes. The results of this study are promising and should the extraction of odours be logistically and financially achievable, then the findings are widely applicable to a broad range of zoos and species. However, without further explanations as recommended above, it would be difficult for many zoos to understand how or why this approach provides an advantage over any other.

Is the rationale for developing the new method (or application) clearly explained?

Partly

Is the description of the method technically sound?

Yes

Are the conclusions about the method and its performance adequately supported by the findings presented in the article?

Partly

If any results are presented, are all the source data underlying the results available to ensure full reproducibility?

Yes

Are sufficient details provided to allow replication of the method development and its use by others?

Yes

Reviewer Expertise:

Zoological animal welfare and behaviour assessment

I confirm that I have read this submission and believe that I have an appropriate level of expertise to confirm that it is of an acceptable scientific standard, however I have significant reservations, as outlined above.
